# Exploring patterns in pediatric type 1 diabetes care and the impact of socioeconomic status

**DOI:** 10.1186/s12916-025-04049-3

**Published:** 2025-04-23

**Authors:** Christopher Nussbaum, Anna Novelli, Amelie Flothow, Leonie Sundmacher

**Affiliations:** 1 Department of Health Economics, School of Medicine and Health, Technical University of Munich, Georg-Brauchle-Ring 60/62, Munich, 80992 Germany; 2 Munich Center for Health Economics and Policy (M-CHEP), Georg-Brauchle-Ring 60/62, Munich, 80992 Germany; 3 Institute for Medical Information Processing, Biometry and Epidemiology (IBE), Faculty of Medicine, Pettenkofer School of Public Health, LMU Munich, Marchioninistraße 15, Munich, 81377 Germany

**Keywords:** State sequence analysis, Clustering algorithms, Type 1 diabetes mellitus, Care pathways, Socioeconomic status, Insurance claims data, Pediatric health data

## Abstract

**Background:**

Managing pediatric type 1 diabetes is complex and requires substantial parental involvement. Adherence to clinical guidelines is often inconsistent, and lower parental socioeconomic status is associated with worse outcomes in affected children. However, few studies have examined these children’s care pathways multidimensionally over time. This study aims to identify latent clusters in the care pathways of pediatric patients with type 1 diabetes mellitus, evaluate guideline adherence and disease management within these clusters, and assess the influence of socioeconomic status on cluster membership.

**Methods:**

We analyzed care pathways for pediatric patients with type 1 diabetes from 2017 to 2019 in the German health system, which provides universal coverage. Using state sequence analysis and clustering algorithms from the TraMineR R package, we identified patient clusters based on healthcare utilization patterns. To assess care quality within these clusters, we compared observed care patterns to clinical guideline recommendations. Our analysis was based on health insurance claims data from Techniker Krankenkasse, a statutory health insurer. From the dataset, which encompassed more than three million patients under the age of 25 years, we derived an age-homogeneous cohort of continuously insured children aged 11 to 14 years with type 1 diabetes in 2017 and extracted relevant healthcare events over a 3-year period.

**Results:**

Based on care patterns, we identified two clusters of children, which we designated as the “guideline-adherent” and “care-with-gaps” clusters. Roughly 25% of our cohort (*n* = 890) fell into the latter cluster, consistently receiving care that fell short of guideline recommendations. For example, these patients had less than half as many quarters with hemoglobin A1c measurement. Lower parental educational attainment and unemployment were predictors of this suboptimal care. We also found that the average number of hospitalizations per child was almost 40% higher in the cluster with less guideline-adherent care.

**Conclusions:**

Despite universal health coverage and frequent contact with the outpatient healthcare system, a substantial proportion of pediatric type 1 diabetes patients in Germany experience suboptimal care, particularly in glycemic diagnostics and screening for complications, leading to worse health outcomes. Higher socioeconomic status is associated with care that more closely adheres to clinical guidelines.

**Supplementary Information:**

The online version contains supplementary material available at 10.1186/s12916-025-04049-3.

## Background

Approximately nine million individuals worldwide live with type 1 diabetes mellitus (T1DM) [1]. The prevalence of T1DM continues to rise, and it remains a major cause of blindness, kidney failure, heart attacks, stroke, and lower limb amputation [1]. Although routine screening and treatment for complications can delay, mitigate, or even prevent these outcomes [1], managing pediatric T1DM is particularly complex. It often requires substantial parental involvement, including frequent blood glucose monitoring, strict oversight of unpredictable dietary and physical activity patterns, and adjustments to medication regimens [2–4]. The simultaneous physiological, cognitive, behavioral, and socio-emotional development of these children further complicates disease management [2, 5].

Clinical guidelines are available in many countries to assist patients, parents, and healthcare providers (HCPs) in navigating the multidimensional care required for T1DM [6–10]. However, research shows that adherence to these guidelines remains inconsistent internationally [11–15]. For instance, screening practices vary considerably among patients [12, 16–19]. Research also indicates that many patients fail to achieve adequate T1DM control, and there remains considerable potential to prevent hospitalizations [20–23]. Puberty and early adolescence represent particularly critical periods for T1DM patients, as they involve the gradual transfer of diabetes management responsibilities from parents to children, often leading to a decline in adherence and glycemic control [24–27]. Moreover, studies have shown that lower parental socioeconomic status (SES) is associated with poorer outcomes, including higher hemoglobin A1c (HbA1c) levels, increased body mass index, and more frequent hospitalizations [28–31]. SES may also affect several aspects of T1DM care, such as screening practices, the regularity of blood glucose self-monitoring, and the likelihood of insulin pump use [17, 28]. These findings are consistent with Andersen’s health service use model, which identifies SES as a key predisposing characteristic [32].

To investigate patterns of healthcare utilization in chronic conditions such as T1DM, researchers have increasingly employed state sequence analysis (SSA), an innovative longitudinal method from the social sciences [33–37]. By using SSA in combination with clustering algorithms, it is possible to identify distinct and latent clusters of care pathways in large and comprehensive datasets without relying on predefined outcomes or thresholds [34, 36]. The characteristics of these clusters can then be compared to ideal care as defined by clinical guidelines to identify relevant deviations [34–36]. The inherently explorative, flexible, and holistic nature of this method represents a distinct advantage over alternative approaches, such as statistical testing, regression, or event history analysis [38–41].

We aim to address two research gaps by applying SSA. First, while deviations from guideline recommendations in T1DM care have been documented for single dimensions [11–15], it remains unclear whether these deviations consistently affect the same individuals. Previous SSAs of T1DM care have focused only on different types of HCP contacts without a more comprehensive consideration of the diagnostic procedures undertaken [37]. More broadly, SSA research has typically concentrated on unidimensional approaches, inherently limiting the analysis to a restricted set of healthcare events. By applying multidimensional SSA, we aim to further explore the capabilities of this method [33, 42]. Second, we have limited understanding of the reasons for suboptimal T1DM treatment and how care disparities persist over time. Longitudinal analyses of healthcare utilization patterns in large datasets could provide valuable insights into the origins and persistence of these disparities [37].

Given the complexity of pediatric T1DM management and the deficits observed in individual care dimensions, we hypothesize that (1) multidimensional care pathways for pediatric T1DM patients in Germany deviate from treatment guidelines. Regional shortages of pediatric and primary care providers in Germany may further contribute to these deviations and support this hypothesis [43]. Based on prior research, we also hypothesize that (2) children from higher socioeconomic backgrounds exhibit greater adherence to treatment guidelines and experience fewer hospitalizations [17, 28, 44]. Thus, we anticipate that our analysis will reveal an association between higher SES and clusters of patients who receive more guideline-adherent care. Such an association could result from parents’ ability to navigate the healthcare system more effectively to obtain appropriate care for their children [45]. Indeed, previous studies have shown that parental health literacy, which is negatively correlated with SES, influences health outcomes in children with chronic diseases [46, 47].

In summary, our study aims to (1) identify patterns of healthcare utilization and guideline adherence among children with T1DM by applying multidimensional SSA to German health insurance claims data and (2) investigate the association between SES and the identified patterns.

## Methods

### Data sources

Our analysis is based on routinely collected claims data provided by Techniker Krankenkasse (TK), a statutory health insurer with nationwide coverage in Germany. In 2016, TK insured approximately 10 million people, making it the largest statutory health insurer in Germany [48, 49]. The dataset covers the years 2016 to 2019 and is limited to continuously insured individuals who were younger than 25 years in 2019. It contains information on all reimbursable procedures in the inpatient and outpatient settings, as well as medication, diagnoses, and patient demographics. In Germany, statutory insurance offers family coverage, allowing dependent children to remain insured under a parent’s plan up to age 25 if they are in education or vocational training and have no independent income. Because most of the patients in our cohort satisfy these criteria, we supplemented the dataset with socioeconomic information from the parent holding the family insurance contract [50]. We provide additional details on the selection of socioeconomic variables later in this section.

### Patient population

The final study cohort consisted of patients with a confirmed T1DM diagnosis in 2016 using the ICD-10-GM-2019 code E10. We considered a diagnosis as confirmed if it appeared twice in different quarters in the outpatient setting or once in the inpatient setting. We restricted our analysis to patients aged 11 to 14 years during the first year of the observation period (2017), corresponding to birth years 2003 to 2006, thereby capturing early adolescence and puberty. Furthermore, clinical guideline recommendations in Germany are homogeneous for this age group, and children of this age typically still live at home and attend school, providing a comparable life context [6].

We excluded patients who routinely received care in a hospital outpatient center. We classified patients as hospital outpatients if they received treatment at a hospital outpatient center in at least eight of the 12 quarters of the observation period from 2017 to 2019. This distinction is important from a health system perspective because certain hospitals in Germany are authorized to provide outpatient care to patients with T1DM and bill these services as a quarterly lump sum, unlike office-based specialists in the German outpatient sector, who must itemize each procedure. As a result, we would thus not have been able to track individual procedures for these patients, which could have created the appearance of substantial care gaps despite their frequent contact with the healthcare system.

### State sequence analysis

SSA examines the progression of states over time. In this study, we defined states by combining healthcare events that were clearly represented and well-coded in the routine data, and clinically relevant based on the national treatment guideline for pediatric diabetes and expert medical opinion [6]. We selected six binary-coded events, which we grouped into three dimensions to improve interpretability and visualization [42, 51].

The first dimension, “HCP contact,” comprised one possible event that we tracked quarterly: a reimbursable procedure with a T1DM diagnosis in an outpatient setting performed by a general practitioner (GP), pediatrician, or internist. The German guideline recommends that patients should have at least one outpatient contact every quarter [6]. The second dimension, “Diagnostics,” comprised two possible events tracked and recommended quarterly: (1) HbA1c measurement and (2) prescription of at least 200 blood glucose test strips, as recommended even for patients using continuous blood glucose monitoring systems [6, 52]. The third dimension, “Screening,” comprised three possible events tracked annually: (1) retinopathy screening (recommended annually to biennially), (2) blood cholesterol screening (recommended biennially), and (3) thyroid hormone screening (recommended annually to biennially) [6]. In summary, perfect guideline adherence over the 3-year observation period would require quarterly outpatient visits, including HbA1c measurement and the prescription of at least 200 blood glucose test strips. Additionally, patients would need to undergo two retinopathy screenings, two thyroid screenings, and at least one cholesterol screening. Further details on the operationalization of these procedures can be found in the supplementary materials.

Next, we specified quarterly or annual states by combining events in each dimension. These states indicate which events took place during each time interval and allow guideline adherence to be assessed. The four possible states in the diagnostics dimension were as follows: no diagnostics (N), measurement of HbA1c only (H), a prescription of at least 200 blood glucose test strips only (B), or both a HbA1c measurement and a sufficient prescription of blood glucose test strips (HB) during the respective quarter. Similarly, there were two possible states in the HCP contact dimension and eight in the screening dimension.

We then obtained state sequences for each dimension and patient by sequentially combining these states over the observation period (2017–2019). Based on these sequences, we calculated pairwise distance matrices using the longest common subsequence (LCS) method. LCS measures the commonality between sequences by counting the number of states that occur in the same order without requiring that they occupy consecutive positions [53, 54]. Following the approaches proposed by Vanasse et al. [42] and Gabadinho et al. [59], we applied min–max normalization to each distance matrix individually before summing them to create a pooled distance matrix across all three dimensions. This approach mitigates skewed results that might arise because the dimensions observed quarterly naturally have a larger possible maximum distance than those observed annually.

Based on the pooled distance matrix, which encompasses pairwise distances between all sequences, we identified clusters using the partitioning around medoids (PAM) clustering algorithm [38]. Drawing on the work of Studer et al. (2013), we determined the optimal number of clusters based on cluster quality criteria and visual inspection of the clustering results. We employed the following approaches that were the most relevant for our analysis: average silhouette width (ASW), point biserial correlation, and Hubert’s C index [38, 55–58]. We compared results for two to 10 clusters [38].

We visualized clusters using state distribution plots and sequence frequency plots [59].

### Covariates and statistical analysis

We calculated descriptive statistics to compare patient-level differences between clusters. To examine differences, we used *t*-tests for continuous variables and chi-square or Kruskal–Wallis tests for categorical variables. Statistical significance was assessed at a 5% significance level.

We used logistic regression to assess the association between SES and cluster membership while controlling for potential confounding factors. We report the results as odds ratios (ORs) and 95% confidence intervals (CIs).

In line with the healthcare utilization literature, we operationalized children’s SES using two parental indicators—occupation and education—and additionally considered a recent family history of migration [32, 60–63]. We categorized parental occupational status (including unemployment) at the beginning of the observation period into four categories based on classifications from the German Federal Employment Agency: (1) unskilled or semi-skilled activities, (2) specialist activities, (3) complex specialist activities, and (4) highly complex activities [28, 64]. Occupation can reflect social standing and may provide access to certain privileges [61, 65]. We measured educational attainment as a categorical variable, distinguishing between the attainment of a university entrance diploma (German “Abitur”) and a university degree [28, 63]. Individuals with higher educational levels are thought to be more receptive to health education messages and may be more able to communicate with HCPs and access health services [61, 66]. A recent family history of migration to Germany could similarly affect health literacy through factors such as language barriers [46, 67] and has indeed been associated with worse T1DM outcomes in children [28]. We defined a recent family history of migration as the parent holding the insurance contract not having German citizenship [28].

We selected the additional covariates based on their documented relevance to healthcare utilization and treatment adherence in T1DM, with a focus on pediatric care. We thus included general patient characteristics (age, gender) as previous research suggests that female and younger patients are more likely to receive optimally adherent care [15]. We also controlled for the following comorbidities commonly associated with T1DM: asthma, autoimmune diseases (e.g., thyroiditis and coeliac disease), cardiovascular diseases (e.g., obesity, hypertension, and hyperlipidemia), and psychological disorders (e.g., depression, personality disorders, and attention-deficit hyperactivity disorder) [6, 68]. Research has shown that concordant comorbidities, i.e., illnesses that overlap with diabetes in pathogenesis or management, can result in similar or better care, whereas discordant comorbidities are associated with reduced quality of care [68].

We also considered an independent variable reflecting healthcare utilization patterns: enrollment in a disease management program (DMP). Enrollment in a DMP is important because it may be associated with a higher frequency of visits and a higher probability of receiving T1DM-specific treatment [69].

Lastly, we controlled for regional variation because need-adjusted healthcare utilization and care patterns have been shown to vary between districts and states in Germany and other countries [15, 70, 71]. To account for this, we included the four district types defined by the Federal Office for Building and Regional Planning: large city, urban district, rural agglomeration, and sparsely populated area [72].

To assess care quality and disease management, we defined two outcome variables: a binary indicator for whether a patient had at least one hospitalization with T1DM as the primary diagnosis and a count variable representing the total number of such hospitalizations during the observation period.

All analyses were performed with R, including the TraMineR and stargazer packages [59, 73].

## Results

### Cohort characteristics

A total of 890 patients met the inclusion criteria. Table 1 presents their characteristics. The children were relatively evenly distributed across the birth years 2003 to 2006, with 48% being female. Approximately half of the cohort (47.8%) was enrolled in a T1DM DMP, and almost one-third had a confirmed diagnosis of a psychological disorder. Socioeconomic data were missing for at least one variable in 16% of the cohort; these patients were included in the clustering analysis but excluded from the regression analysis. The distribution of district types was consistent with the German average, although a higher proportion of children lived in urban districts [74]. The proportions of parents with a university entrance diploma, a university degree, and unemployment were 44.6%, 31.1%, and 5.6%, respectively. The majority of parents (45.4%) worked in specialist occupations. These proportions are representative of all parents of children in the respective birth year cohorts with available socioeconomic data from TK, with only a slightly higher proportion (5.8%) of unemployed parents in the full dataset (*n* ≈ 280,000).

**Table 1:** Description of patient population stratified by identified clusters and sub-groups

	Total	Cluster 1: guideline-adherent	Cluster 2: care with gaps	***p* v**alue
	***n* = **890	***n* = **671	***n* = **219	
Year of birth				0.025
2003	27.9%	29.7%	22.4%	
2004	27.4%	27.4%	27.4%	
2005	23.0%	22.4%	25.1%	
2006	21.7%	20.6%	25.1%	
Sex (female)	48.0%	48.1%	47.5%	0.929
Participation in disease management program	47.8%	53.1%	31.5%	0.000
Autoimmune comorbidity	14.2%	15.4%	10.5%	0.094
Asthma comorbidity	9.2%	9.7%	7.8%	0.471
Psychological comorbidity	30.6%	31.1%	28.8%	0.562
Cardiovascular comorbidity	10.4%	11.2%	8.2%	0.265
Socioeconomic data missing	16.4%	17.1%	14.2%	0.352
University entrance diploma (Abitur)	44.9%	47.3%	37.8%	0.046
University degree	31.9%	32.6%	29.8%	0.496
Job type				0.590
Un-/semi-skilled activities	7.2%	6.6%	9.0%	
Specialist activities	45.0%	44.5%	46.3%	
Complex specialist activities	19.5%	19.6%	19.1%	
Highly complex activities	28.3%	29.3%	25.5%	
Recent family history of migration	5.9%	5.8%	5.9%	0.978
Unemployment status	5.1%	3.9%	8.6%	0.026
Place of residence (district type)				0.168
Large city	24.6%	24.7%	24.4%	
Urban district	47.4%	46.0%	51.7%	
Rural agglomeration	14.1%	15.7%	9.1%	
Sparsely populated area	13.9%	13.5%	14.8%	
Outcome variables				
Patients with at least one hospitalization	57.6%	55.9%	63.0%	0.076
Average number of hospitalizations per child	1.22	1.11	1.54	0.004

To evaluate the statistical significance of differences between clusters, we calculated *p* values using *t*-tests for continuous variables and chi-square or Kruskal–Wallis tests for categorical variables

### Cohort-wide healthcare utilization

**Fig. 1:**
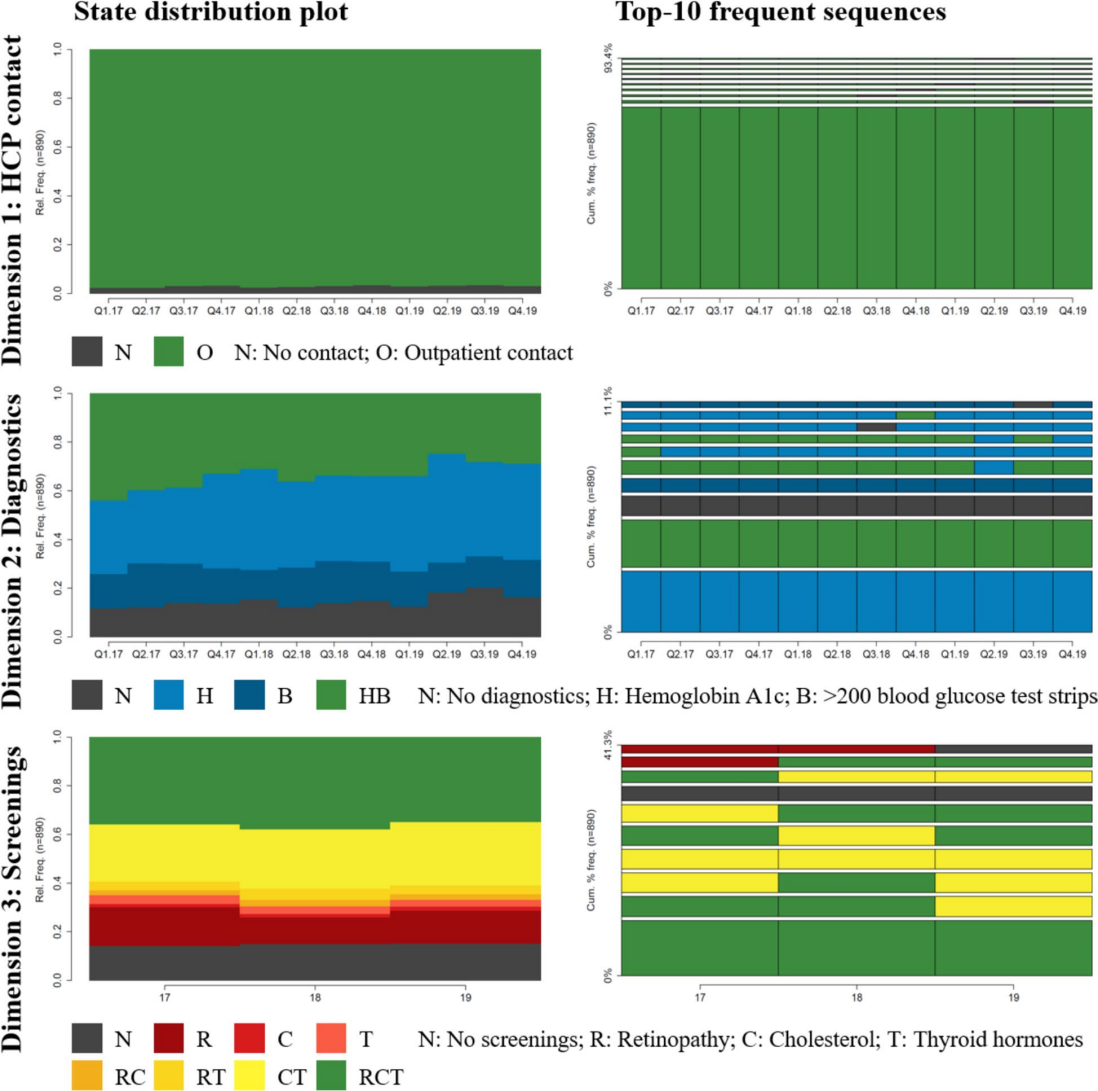
Visualization of states and state sequences per dimension. Note: We use state distribution plots for the visualization of states and top 10 most frequent sequences for the visualization of sequences per dimension

Figure 1 displays the most common states per time interval across all patients, as well as the 10 most frequent state sequences. The first dimension (“HCP contact,” tracked quarterly) shows that over 95% of patients had the recommended outpatient contact (“O”) with a T1DM diagnosis in any given quarter. The most common sequence consisted of 12 consecutive quarters with a T1DM-related outpatient contact. The second dimension (“Diagnostics,” tracked quarterly) shows that over 70% of patients underwent the recommended HbA1c measurement, either with (“HB”) or without (“H”) a prescription of blood glucose test strips, in any given quarter. Although the two most common sequences consisted of 12 consecutive “H” or “HB” states, this dimension showed more variability, with the top 10 sequences accounting for only 11% of the cohort compared to roughly 93% for the first dimension. The third most common sequence was “Non-diagnostics.” The third dimension (“Screenings,” tracked annually) shows that roughly 85% received at least one screening per year. The most frequent state was the one in which all three screenings were performed. The most common sequence consisted of three consecutive years with all recommended screenings, whereas three consecutive years without any of the recommended screenings was the seventh most common sequence.

### Cluster analysis

**Fig. 2:**
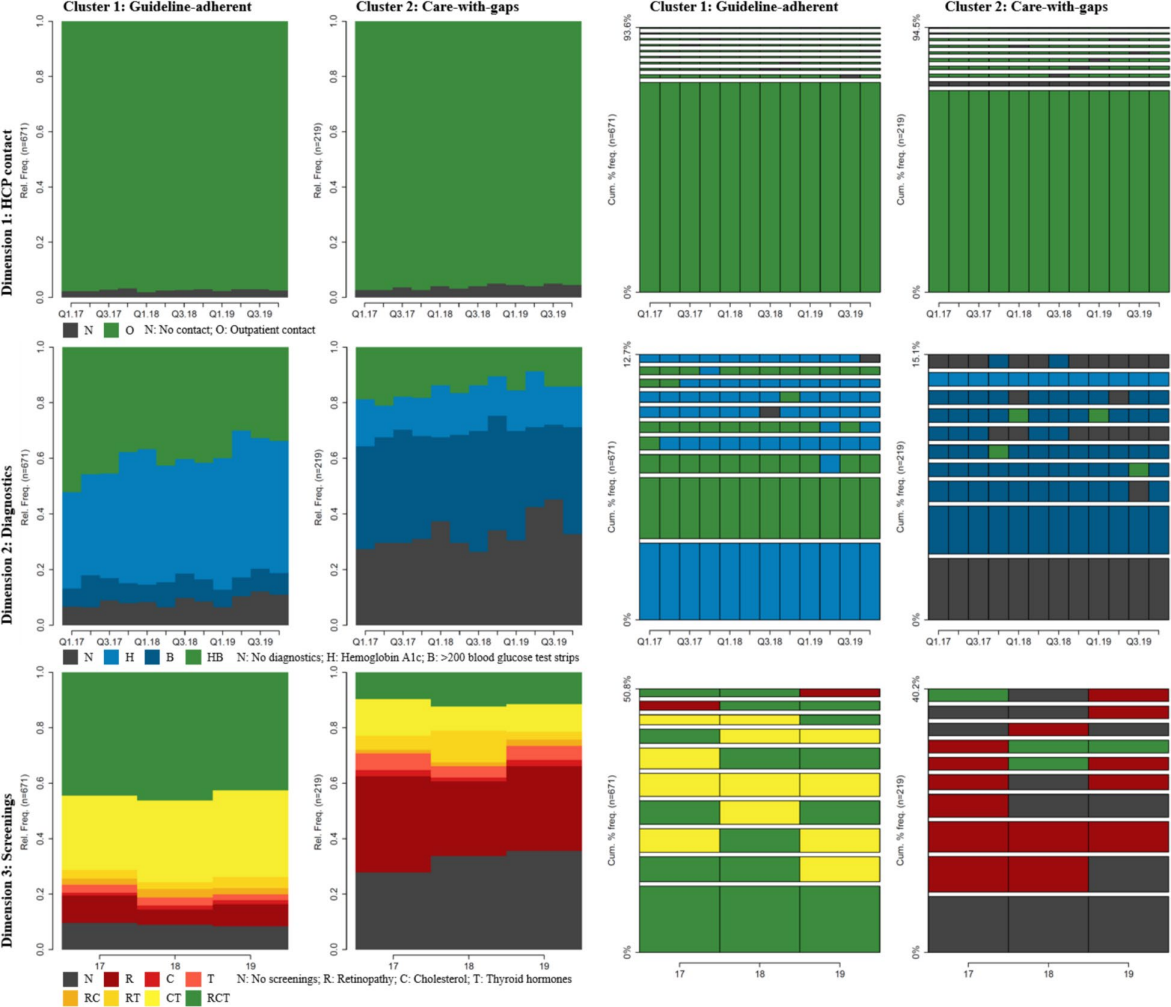
Visualization of states and state sequences per dimension and cluster. Note: We use state distribution plots for the visualization of states and top 10 most frequent sequences for the visualization of sequences per dimension and cluster

Next, we present the clustering results. The algorithm identified two clusters as the optimal result based on our cluster quality criteria (Fig. 2, ASW 0.22). Patients in cluster 1 received care that was generally consistent with guideline recommendations across all dimensions, leading us to designate it as the “guideline-adherent” cluster. In contrast, patients in cluster 2 received care that fell short of treatment guideline recommendations, leading us to designate it as the “care-with-gaps” cluster. The guideline-adherent cluster consisted of 671 (75%) patients, and the care-with-gaps cluster consisted of 219 (25%) patients.

The two clusters differed mainly in the diagnostics and screening dimensions. In the diagnostics dimension, states containing the HbA1c measurement accounted for roughly 85% of all states in any given quarter for patients in the guideline-adherent cluster. In the care-with-gaps cluster, this figure dropped to approximately 35%, meaning that many patients in this cluster either did not undergo any diagnostic procedures or received only prescriptions for blood glucose test strips. In the screenings dimension, the care-with-gaps cluster was dominated by states with either no screenings or only one screening per year (accounting for roughly 70% of states in any given year). In contrast, roughly 80% of patients in the guideline-adherent cluster received at least two screenings per year. The two clusters differed only slightly in the HCP contact dimension, with the care-with-gaps cluster having slightly more quarters without contact with the outpatient healthcare system.

### Cluster background

The characteristics of children and their parents differed between clusters, exhibiting distinct patterns (Table 1). At the 5% significance level, year of birth, DMP enrollment, parental attainment of a university entrance diploma, and parental unemployment status were significantly associated with cluster membership in unadjusted analyses. Parents of patients in the care-with-gaps cluster had lower educational attainment and were more often unemployed compared to parents in the guideline-adherent cluster. In the guideline-adherent cluster, patients were slightly older and more frequently enrolled in a DMP. Although the difference was not significant, comorbidities, especially autoimmune diseases, were slightly more prevalent in the guideline-adherent cluster, and their parents were more likely to work in higher-skilled occupations.

Both outcome variables indicate that patients in the care-with-gaps cluster were less successful in avoiding hospitalizations. On average, these patients had 39% more hospitalizations than those in the guideline-adherent cluster (*p* = 0.004). Although this difference was not significant (*p* = 0.076), the proportion of children with at least one preventable hospitalization was seven percentage points higher in the care-with-gaps cluster.

Using logistic regression, we examined the association between patient characteristics and cluster membership at the 5% significance level. The results are presented in Table 2. Overall, the findings suggest that there is a significant association between SES and cluster membership. Compared to the care-with-gaps cluster, having unemployed parents (OR: 57%) decreased the odds of belonging to the guideline-adherent cluster. In contrast, being enrolled in a DMP (OR: 142%) increased the odds of being in the guideline-adherent cluster. While having parents with a university entrance diploma (OR: 39%) increased the odds of being in the guideline-adherent cluster, this association was not significant at the 5% level.

**Table 2:** Results from logistic regression model predicting the likelihood of cluster membership

Factor	Dependent variable
	Cluster 1: guideline-adherent
	OR (95% conf. interval)
Year of birth, mean centered	0.875* (0.720, 1.029)
Participation in disease management program	2.428*** (2.076, 2.780)
Autoimmune comorbidity	1.695* (1.159, 2.231)
University entrance diploma (Abitur)	1.387* (1.037, 1.736)
Unemployment status	0.433** (− 0.337, 1.203)
Constant	1.734*** (1.468, 2.001)

These are results from a logistic regression with cluster membership as the outcome. We use odds ratios (ORs) to quantify the association between predictor variables and the likelihood of membership the guideline-adherent cluster relative to the base outcome (care with gaps cluster). An OR greater than 1 indicates a higher likelihood of being in the specified cluster compared to the base outcome, whereas an OR less than 1 indicates a lower likelihood. The significance levels are as follows: *p < 0.1; **p < 0.05; ***p < 0.01. Confidence intervals for the ORs are provided to indicate the precision of the estimates

### Exploratory analyses

Additional exploratory analyses of the 139 excluded hospital outpatients, not reported in the appendix, suggest that these children had the lowest proportion of DMP enrollment and the highest prevalence of asthma and cardiovascular conditions. They were also more likely to have a recent family history of migration to Germany and to live in urban areas.

## Discussion

We conducted a multidimensional SSA on a large and comprehensive dataset of health insurance claims from Germany to identify healthcare utilization patterns and assess adherence to clinical guidelines in the treatment of pediatric type 1 diabetics. In addition, we investigated the association between these patterns and SES. By applying an unsupervised machine learning algorithm to nationwide data covering 3 years, we identified two distinct clusters: a “care-with-gaps” cluster, which comprised one-fourth of the study cohort and was characterized by substantial deficiencies in T1DM care, and a “guideline-adherent” cluster, which comprised the remaining three-fourths and received higher-quality care that more closely corresponded to the relevant clinical guideline recommendations.

This finding confirms our hypothesis regarding the presence of clusters of patients who consistently receive suboptimal care across multiple care parameters. While over 95% of patients in both clusters had contact with the healthcare system in any given quarter, the care-with-gaps cluster showed marked deficiencies, especially in glycemic testing and screening for diabetic retinopathy, thyroid disorders, and hypercholesterolemia. Despite having only marginally more quarters with outpatient contact, the guideline-adherent cluster had more than twice as many quarters with HbA1c measurements and at least two screenings compared to the care-with-gaps cluster.

Our finding that 75% of patients belonged to the guideline-adherent cluster is slightly higher than estimates reported in previous studies [14, 15, 17–19]. For example, Amed et al. [15] found that only 45% of patients achieved care goals. The higher proportion in our study may be attributable to the use of a more flexible clustering algorithm, in contrast to the stringent application of predefined thresholds for the frequency of diagnostic measurements by Amed et al. [15].

For the children in the care-with-gaps cluster, which comprised 25% of the overall study cohort, there is no immediate explanation for the observed care gaps, which persisted despite frequent contact with HCPs. These gaps may result from physicians’ unfamiliarity with clinical guidelines, deliberate deviations from guideline recommendations, or barriers to patient engagement. Although research on guideline awareness in pediatric T1DM is scarce, one study suggests that HCPs in the USA generally possess sufficient levels of knowledge in this context [17]. Whether this holds true for providers in Germany, however, is unclear. A deliberate decision not to follow guideline recommendations may reflect challenges in achieving optimal disease control. Further research, potentially incorporating clinical parameters, is needed to better understand this finding.

Through statistical testing of summary statistics and logistic regression analysis, we found that parental education and unemployment status were significantly associated with cluster allocation, confirming our second hypothesis. Patients whose parents held a university entrance diploma were more likely to be in the guideline-adherent cluster, whereas those with an unemployed parent were more likely to be in the care-with-gaps cluster.

This socioeconomic gradient reflects existing care gaps and may result from disparities in access to specialized care, HCP biases, or patient-initiated demand for procedures. Although Germany’s universal health coverage facilitates access to healthcare, barriers related to accessibility and availability may still persist. [75]. Evidence suggests that factors such as travel time to HCPs and the likelihood of consulting a specialist vary regionally and correlate negatively with the SES of the local population, contributing to inequities in care [70, 76, 77]. Moreover, although clinical guidelines recommend treatment in specialized pediatric diabetology centers [6], socioeconomically disadvantaged patients are more likely to consult non-specialized HCPs and may therefore receive suboptimal care [77]. However, because our dataset does not include information on whether HCPs specialize in diabetology, we could not determine the extent to which the types of providers patients consulted contributed to the observed care disparities. Additionally, previous research has shown that even when treating the same patient population, HCPs may exhibit biases that favor patients with higher SES [78, 79]. Lastly, parents from higher SES groups, who tend to have greater health literacy, may proactively request certain procedures for their children in accordance with clinical guidelines [46].

Our research provides evidence of suboptimal disease management in the care-with-gaps cluster. Although the difference in the proportion of children with at least one hospitalization during the observation period in this cluster was not statistically significant, the average number of T1DM-related hospitalizations was both substantially and statistically significantly higher among these patients. Taken together, these findings suggest that inadequate adherence to guideline-based care may increase the risk of repeated hospitalizations in this population.

Beyond our primary findings, several additional observations offer insights into the broader context of our study cohort. The guideline-adherent cluster tended to be older, more often comorbid with autoimmune diseases, and more frequently enrolled in a DMP. Despite uniform guideline recommendations, the association with older age may reflect that, in practice, screenings are introduced into a patient’s routine care gradually over time rather than immediately upon his or her becoming eligible according to the guidelines. The higher prevalence of autoimmune comorbidities, such as thyroiditis, probably reflects the need for regular thyroid hormone testing required by these conditions and T1DM. Lastly, DMP enrollment appears to incentivize both HCPs and patients to prioritize guideline-adherent care.

Due to billing regulations in Germany, we excluded approximately 140 children who received routine T1DM care in hospital outpatient centers from our cohort. In these settings, hospitals bill outpatient services as a quarterly lump sum rather than itemizing individual procedures, preventing us from accurately assessing their healthcare utilization. Although this billing practice also applies to hospitalized patients, its impact on our findings is smaller due to the relatively low number of hospitalizations in the cohort. In unreported analyses, we found that these children had higher rates of asthma, cardiovascular diseases, and a recent family history of migration. They were also more likely to live in larger cities rather than sparsely populated areas, which is consistent with the location of such centers. Although we cannot draw conclusions about the quality of care provided to hospital outpatients, the higher prevalence of asthma and cardiovascular diseases suggests a need for specialized multidisciplinary care, which is typical of hospital outpatient settings [80]. The higher proportion of patients with a recent family history of migration may reflect the complexity of navigating the healthcare system, with hospitals serving as more accessible initial points of contact. This observation is consistent with studies from Germany and Austria indicating that patients who are migrants or whose parents were born outside of Germany are more likely to lack a GP and seek care directly from hospital outpatient departments without prior GP contact [81, 82].

Our study is the first to apply multidimensional SSA to German insurance claims data. However, it has several important limitations. First, the analysis is based on data collected routinely for billing and reimbursement purposes. Although it is thus comprehensive regarding diagnoses, reimbursable procedures, and prescribed medications, it lacks clinical parameters and most socioeconomic variables. Moreover, the available socioeconomic variables were derived solely from the parent through whom a child was insured and may therefore not fully capture the complexity of a family’s SES. The exclusion of 16% of the cohort from the regression analysis due to missing SES data may have introduced selection bias, potentially affecting the generalizability of our findings. Second, the analysis yielded an ASW of 0.22, suggesting a weak clustering structure based on classical thresholds [38, 83]. However, in comparison to unidimensional SSAs in healthcare research, our ASW falls within the lower half of the observed span of 0.09–0.52 [34, 36, 84–86]. Previous multidimensional SSAs in other healthcare settings have either not reported or not relied on ASW as a quality indicator [42, 87, 88]. Although the ASW score may indicate a weak structure, the sequence visualizations reveal considerable differences between the clusters, suggesting that traditional thresholds may need to be reconsidered for multidimensional SSA. Third, several factors should be considered when generalizing our findings to other settings. Compliance with clinical guidelines varies by country and context. For example, the American Diabetes Association recommends retinopathy screening every 2 years, whereas the German Diabetes Association recommends screening every 1 to 2 years [6, 7, 89]. Additionally, although SES universally affects health outcomes and treatment patterns, the extent and nature of this impact differ across settings. Our analysis is based on data from a single statutory health insurer (SHI), and because SHIs in Germany vary in the socioeconomic composition of their insured populations, the proportion of patients receiving suboptimal treatment in our study may not be representative of the broader T1DM population in Germany. However, compared to countries with less comprehensive health coverage, Germany’s system may facilitate better access to care [90].

Our findings emphasize the importance of continuously disseminating clinical guidelines among practitioners to improve adherence, particularly given the frequent interactions that T1DM patients in Germany have with healthcare services. Policies that incentivize guideline adherence, such as the broader implementation of DMPs, could further improve T1DM care. Additionally, providing families from lower socioeconomic backgrounds with comprehensive information on key screening components may empower them to actively request patient-centered care, thereby supporting adherence.

## Conclusions

By employing SSA and unsupervised machine learning, we investigated the healthcare of pediatric T1DM patients in Germany over a 3-year period in a study cohort derived from nationwide insurance claims data. We found that one in four children with T1DM consistently received care that fell short of clinical guideline recommendations across multiple care dimensions. Logistic regression analysis revealed that membership in the less guideline-adherent care cluster was associated with lower SES. Future research should further explore the potential of multidimensional SSA, including the integration of clinical parameters, and aim to better understand the underlying mechanisms of the observed socioeconomic gradient.

## Supplementary Information

Supplementary Material 1.

## Data Availability

The data that support the findings of this study are available from the statutory health insurance Techniker Krankenkasse but restrictions apply to the availability of these data, which were used under license for the current study, and so are not publicly available. Data are however available from the authors upon reasonable request and with permission of Techniker Krankenkasse and its supervisory authority.

## Abbreviations

ASW: Average silhouette width
CI: Confidence intervals
DMP: Disease management program
GP: General practitioner
HbA1c: Hemoglobin A1c
HCP: Healthcare providers
LCS: Longest common subsequence
OR: Odds ratios
PAM: Partitioning around medoids
SES: Socioeconomic status
SHI: Statutory health insurance
SSA: State sequence analysis
T1DM: Type 1 diabetes mellitus
TK: Techniker Krankenkasse (German SHI)
