# Supplementary material for: Exploring patterns in pediatric type 1 diabetes care and the impact of socioeconomic status
Source: BMC Med. 2025 Apr 23;23:229. doi: 10.1186/s12916-025-04049-3 (PMC12016072; doi:10.1186/s12916-025-04049-3)
Supplement: Supplementary file 1 — Supplementary Material 1. [file 12916_2025_4049_MOESM1_ESM.docx]

**Supplemental material: Exploring Patterns in Pediatric Type 1 Diabetes Care and the Impact of Socioeconomic Status –** Operationalization of diagnoses and healthcare events in routine data

In the following, we present the codes to identify type 1 diabetes patients and relevant procedures in German insurance claims data:

- Diagnosis: ICD-10-GM-2019 code E10
- Relevant co-morbidities
  - Asthma: ICD-10-GM-2019 code J45
  - Autoimmune diseases: ICD-10-GM-2019 codes K900, E063
  - Cardiovascular: ICD-10-GM-2019 codes E66, E78, I10
  - Psychological: ICD-10-GM-2019 codes F32, F33, F34, F38, F39, F40, F41, F43, F45, F50, F60, F68 ,F69, F84, F90
- Relevant outpatient contact:
  - ICD-10-GM-2019 codes E10 and E14 given that patients were identified initially with E10 code
  - Restricted to type of health care practitioner filtering only for family doctors, pediatricians, and internists, i.e. identified by German “Facharztgruppe”: “01 Allgemeinmediziner (Hausarzt)”, “02 Arzt/Praktischer Arzt (Hausarzt)”, “03 Internist (Hausarzt)”, “34 Kinderarzt (Hausarzt)”, “40 Kinderarzt (Facharzt)”, “46 Kinder- und Jugendmedizin mit Schwerpunkt und Teilnahme an haus- und fachärztlicher Versorgung”
- Relevant inpatient stay: ICD-10-GM-2019 codes E10 and E14 as main diagnosis given that patients were identified initially with E10 code
- Procedures:
  - HbA1c diagnostic: EBM-code 32094
  - Fundoscopy: EBM-codes 06333, 99749E, 99349
  - Lipid/cholesterol screening: EBM-codes 32882, 32060, 32061, 32062, 32063
  - Thyroid hormone assay: EBM-codes: 32101, 32320, 32321
- Prescriptions:
  - Blood glucose test strip prescriptions: ATC-code V04CA03
